# Metagenomic Analysis of Gut Microbiome in Gout Patients with Different Chinese Traditional Medicine Treatments

**DOI:** 10.1155/2022/6466149

**Published:** 2022-10-10

**Authors:** Jingjing Xie, Jing Wang, Fang Zhao, Xia Qiu, Jianwei Chen, Yangyang Jia, Panpan Qin, Yuanfang Zhu, Jianyong Zhang, Binbin Wang

**Affiliations:** ^1^The Fourth Clinical Medical College of Guangzhou University of Chinese Medicine, Shenzhen, Guangdong, China; ^2^Department of Rheumatism, Shenzhen Traditional Chinese Medicine Hospital, Shenzhen, Guangdong, China; ^3^Department of Medical Genetics and Developmental Biology, School of Basic Medical Sciences, Capital Medical University, Beijing 10069, China; ^4^BGI-Qingdao, BGI-Shenzhen, Qingdao 266555, China; ^5^Qingdao-Europe Advanced Institute for Life Sciences, BGI-Shenzhen, Qingdao 266555, China; ^6^Center for Genetics, National Research Institute for Family Planning, Beijing, China

## Abstract

**Introduction:**

Changes in eating habits have made gout a metabolic disease of increasing concern. Previous studies have indicated that there are significant differences in species composition and abundance of gut microbiome in gout patients compared with average. Considering that traditional Chinese medicine has a momentous effect in treating gout, the research study aimed to explore the differences of genomic and metabolomics of gut microbiome before and after traditional Chinese medicine treatment in patients with gout.

**Method:**

30 patients with gout and 29 matched controls were recruited of which 16 patients took H treatment and 14 patients took T treatment. Stools were collected twice for patients before and after treatment and only once for controls. A total of 89 samples were annotated with metagenomic species and functions, and the enrichment analysis of differential genes and KO pathway was carried out.

**Result:**

The results showed a decrease in the diversity of gut microbiome in gout patients and the gene abundance and metabolomics had great differences among study groups. The number of bacterial genera also had significant differences among treatment groups. Moreover, among different groups, the regulation of different species was variously correlated. The correlation between species and clinical laboratory indicators in the rising group was stronger than that in the decreasing group and the upregulation of some strain was related to the content of urea nitrogen.

**Conclusion:**

After the traditional Chinese medicine treatment, the glutathione pathway was significantly enriched and some pathogenic bacteria were significantly inhibited. The study suggests that traditional Chinese medicine treatment may exert its therapeutic effect by inhibiting relevant pathways.

## 1. Introduction

Gout is a metabolic disease of urate crystal deposition, which is related to chronic hyperuricemia [1]. Environmental factors such as eating habits can cause hyperuricemia including red meat, seafoods, alcohol, or fructose were associated with an increased risk of developing gout [2]; R. [3], and genetic factors also play an important role. Compared with healthy people, the content of uric acid in the serum increases, which is largely related to the poor excretion of uric acid by the kidneys and intestines [4]. When the gout patient has a single joint inflammation reaction during the acute attack (“gout attack”), such as severe pain in the big toe [5], treatment is required to ensure the reduction or disappearance of the acute symptoms. That is to say, when gout attacks, what we do first is not to cure gout, but to reduce uric acid and anti-inflammatory symptoms in the acute phase or acute remission phase to reduce the patient's pain. If the patient with gout does not take the medicine during the acute onset period, it will definitely not get better in 2 weeks. The clinical symptoms shown are that the uric acid cannot be lowered and the siltation of the joint cavity of patient is getting more and more serious. The commonly used medication is “treatment targeting serum uric acid,” which reduces serum uric acid [4]. In addition to the commonly used febuxostat and allopurinol [6, 7], traditional Chinese medicine(TCM) has been proved to be effective for GA prevention and treatment with the advantage of Chinese medicine according to different syndromes and the stages of disease, the treatment methods of TCM will also greatly reduce the patient's suffering from the clinical effect, which can be clearly seen that patients with gout have reduced their joint swelling (W. H. [8]; P. [9]).

There have been many advances in the research on the pathogenesis of gout and the effect of drugs, no matter from the biochemical indicators, molecular mechanism, or omics including genomics, transcriptomics, epigenomics, and metabolomics. Research on clinical laboratory indicators of gout has given clinical insights into the pathophysiology of hyperuricemia and gouty arthritis. Studies have shown that during the development of gout, the closely related monosodium urate (MSU) crystals can affect the production of certain immune cells, cytokines, and effector molecule expression, triggering both innate and adaptive immune responses [10]. The development of the genomics has brought about the study of gout disease at the genome level, using meta-analysis first to reveal the loci associated with crystal-induced inflammation which is the last step in gout development [11]. In order to study the epigenetic background of gout inflammation independent of hyperuricemia and its relationship with heredity, this article conducted co-methylation analysis and functional localization of the identified loci and analysis of DNase hypersensitivity and histone markers, together with the transcription factor mapping identified several novel genes specific to gouty inflammation [12].

Metagenomics with gut microbes, as a major perspective in the study of diabetes [13], cancer [14], and mental diseases [15] in recent years, has also been used in the study of arthritis diseases [16, 17]. Since the gastrointestinal tract eliminates one-third of the uric acid load [18], research on the gut microbiota and metabolic abnormalities of patients with gout is also attracting attention. 16S analysis provided us a way to investigate the relationship between the primary gout and the gut microbiota, for example, one study with result that the diversity of both *Bacteroides* and *Clostridium* in patients with primary gout and the difference from that of normal individuals [19]. Other study found that the *Proteobacteria* phylum and the *Escherichia-Shigella* genus were more abundant in patients with tophaceous gout than in controls, and some core microbiota of both gout groups might perform functions linked to one-carbon metabolism, nucleotide binding, amino acid biosynthesis, and purine biosynthesis [20]. Besides, combined analysis of 16S and metabolome by fecal samples suggested us that it may effectively characterize diseases [21]. Compared to 16S analysis, metagenomics analysis could find that the intestinal microbiota of gout patients was highly distinct from healthy individuals in both organismal and functional structures, even build up a diagnosis model as a possible future application approach, higher than the blood-uric-acid based approach [22].

However, we found that previous studies on the gut microbes of patients with gout, whether using16S rRNA or metagenomics, were limited to the difference in the composition and abundance of the species between gout patients and healthy controls, little using groups of patients before and after medication to study the changes in gut microbes. If one case group is changed into two case groups adding before and after medicine for gout patients in the analysis, and the control group using healthy people is compared at the same time, it will be more clear to find differences about the effect of the medicine on the gout patients. The purpose of this article is to compare patients with gout before and after medication and with healthy controls and find consistent trends regardless of gene or species annotation or KEGG function.

## 2. Materials and Methods

### 2.1. Sample Collection

We recruited 30 gout patients and 29 healthy age-matched controls from Shenzhen Traditional Chinese Medical Hospital. All patients recruited in the present study only suffered from gout, and carriers of any other diseases were excluded from this study. The years of onset are shown in Table S1 (6.33 ± 5.58 years, mean ± SD). These 30 gout patients were treated with different traditional Chinese medicine H named Hushen Tongfengtai and T named Tongfengtai for two weeks, and fecal samples were collected before the medication named A group and after the medication named B group, and total 60 patient samples were taken. All recruited people including gout patients and healthy controls were excluded from other medication. Among them, the sampling standard for gout patients after taking the medicine was significant released or disappearance of gout symptoms, for example, the joints swelling of gout patients was released. One fecal sample was collected from each healthy control and a total of 29 control samples were obtained. All total 89 fecal samples were used in our study finally. All the enrolled personnel have been informed and voluntarily signed the informed consent form, and the sampling was also carried out after obtaining the ethical approval from the Ethics Committee of Shenzhen Traditional Chinese Medical Hospital.

### 2.2. Metagenomics Library Construction and Sequencing

Total DNA was extracted from each of the samples and quality checked. An amount of 0.5 *μ*g high-quality DNA was used for library construction using the MGIEasy DNA Rapid Library Prep Kit (BGI, catalog no, 1000006985) following the manufacturer's instructions. Briefly, DNA was randomly fragmented using a Covaris LE220 ultrasonicator (Covaris, Woburn, MA, USA) and DNA fragments ranging from 200 to 400 bp were selected and purified with AMPure XP magnetic beads. Selected DNA fragments were then subjected to PE index ligation and ligated DNA was purified. The purified ligated DNA was subjected to form circularized DNA and subsequent rolling circle amplification (RCA) to generate DNA nanoballs (DNBs) and the DNBs were then sequenced at the DNBSEQ-T1 platform generating PE100 reads.

### 2.3. Metagenomics Species and Functional Annotation

In order to obtain the accurate and reliable results, the low-quality reads were filtered out to obtain clean data by SOAPnuke (v1.5.6) with parameters −l 20 −q 0.2 −n 0.05, and then the human host contamination was removed by using Bowtie2 (v2.2.5) with 90% similarity [23] to generate the high-quality clean data for downstream analyses. Then, the reads with high quality from each sample were aligned against Integrated Gene Catalog (IGC, v100064) (J. [24] by Bowtie2 (v2.2.5) with parameters --sensitive --mp 1,1 --np 1 --score-min L,0,-0.1. Moreover, we used Salmon (v0.9.1) [25] to calculated the gene abundance for each sample. Similarly, based on the previous species and functional annotation in IGC database, we retrieved proteins by matching gene ID and obtained annotation results and generated the species, KO and pathway abundance profiles. For biodiversity analysis, the Shannon index was usually used on alpha diversity to evaluate the community diversity of samples. The Bray–Curtis distance and the Jaccard distance were used on Beta diversity to measure the differences between the samples. All the *α*- and *β*-diversity statistics were performed by using “vegan” package of R software (v3.6.1).

### 2.4. Statistical Analysis

The nonparametric test method Wilcoxon rank-sum test and Kruskal–Wallis test were used for the differential gene, KO and species analysis [26], while Student's *t*-test and Wilcoxon rank-sum test was used to conduct nonparametric tests on the two groups of independent samples and determine whether there was a difference between two groups, and Kruskal–Wallis test was used to test the difference between three or more groups of samples. Moreover, the *p* value correction is carried out by *p* adjust in the R package, and the correction method is “BH” (i.e., Benjamini-Hochberg). The correlation analysis of the gut microbiomes and clinical laboratory indicators were preformed using the Spearman algorithm, and only the robust correlations with |*r*| > 0.40 and *P* < 0.05  were considered. The correlation was calculated and visualized by R software (v3.6.1).

## 3. Results

### 3.1. Basic Data Analysis

In our study, 30 gout patients and 29 healthy controls were recruited. 16 gout patients took H traditional Chinese medicines, and other 14 patients took T traditional Chinese medicines. For each patient, fecal samples were collected before and after the medication, and we obtained total 60 fecal samples for patients including 16 HA and 16 HB samples and 14 TA and 14 TB samples. For each healthy control, one fecal sample was collected. Total 89 fecal samples were used finally with different groups displayed according to different classification standards (Table 1). After DNA extraction, library construction and sequencing, a total of 2.06 TB data were generated for 89 samples, with average 22.44 GB for each sample. Moreover, after filtering out the low quality and duplication reads, the clean reads Q30 were about 92.37%, with the clean data ratio more than 91.50% (Table S1). These indicators reflected that our data were of high-quality and enough for subsequent analysis.

The clean reads were mapped to the human gut microbiome integrated gene catalog (IGC) (J. [24] to generate the gene abundance profile. A total of 5,195,879 nonredundant genes were identified in 89 samples, with an average comparison rate of 75.15%, indicating that our data can be effectively compared to the gene set (Table S1). We summarized the number of genes annotated at the phylum level and the proportion of all genes through the IGC species annotation result and found that the *Firmicutes* (54.48%), *Bacteroidetes* (25.24%), *Proteobacteria* (12.29%), *Actinobacteria* (5.40%), *Fusobacteria* (1.11%), and *Verrucomicrobia* (0.66%) have the highest proportions (Table S2). Combined the previously published research studies about Asian gut microbiota, we found that three phyla, *Firmicutes*, *Bacteroidetes*, and *Proteobacteria*, were the most abundant species. According to the functional annotation results of IGC database, 9,073 KOs were detected by matching the gene ID. Then, we generated the species, KO, and pathway abundance profiles by the gene abundance profile and then performed the different analysis between groups.

### 3.2. Comparison of Differences between Groups

**Figure 1:**
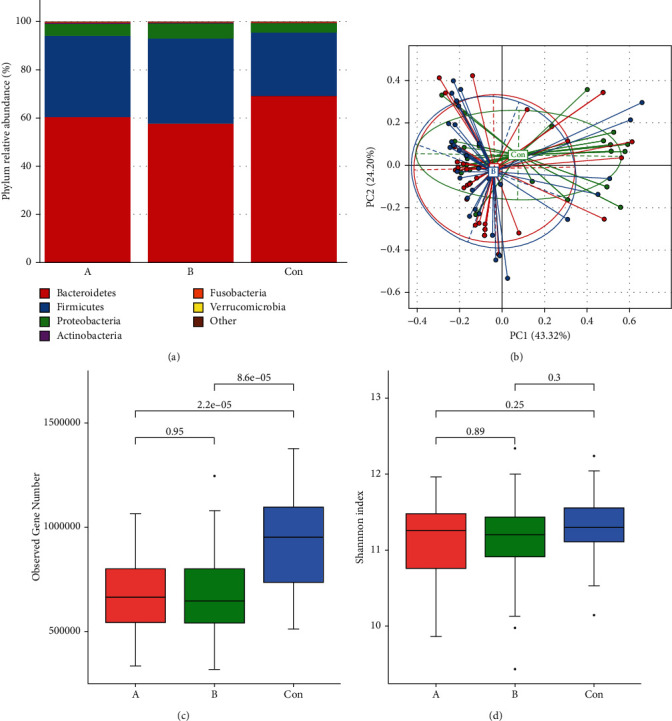
The fecal microbiome of gout patients compared to healthy controls. (a) The species annotation results of the three groups at the phylum level; (b) PCoA of the three groups, reflecting the similarity between groups; (c, d) Box plots reflecting alpha diversity.

We calculated the relative abundance in group A and B and Con in the two taxonomic levels of phyla and genus, respectively. Among them, *Firmicutes*, *Bacteroidetes*, and *Proteobacteria* with the highest proportion of genes were still the most abundant in three groups (Figure 1(a) and Table S2). PCoA (Principal Co-ordinates Analysis) is often used in microbial *β*-diversity analysis, by entering the sample similarity distance table. We found that the distinction between group A and B was not large, and the distinction between group A vs Con or between group B vs Con was more obvious by comparing group A and B and Con (Figure 1(b)), indicating that the gut microbiota of gout patients and nongout patients were different, but the effect on the gut microbiota between medication before and after was not significant. As for the box plot of *α*-diversity analysis, we found that there was no significant difference between group A vs B, but the observed gene richness of group A vs B was significantly lower than group Con, regardless without significantly results of the Shannon index (Figures 1(c) and 1(d)). It indicated that the gut microbiota diversity of gout patients (group A vs B) was lower than the healthy control samples (group Con).

**Figure 2:**
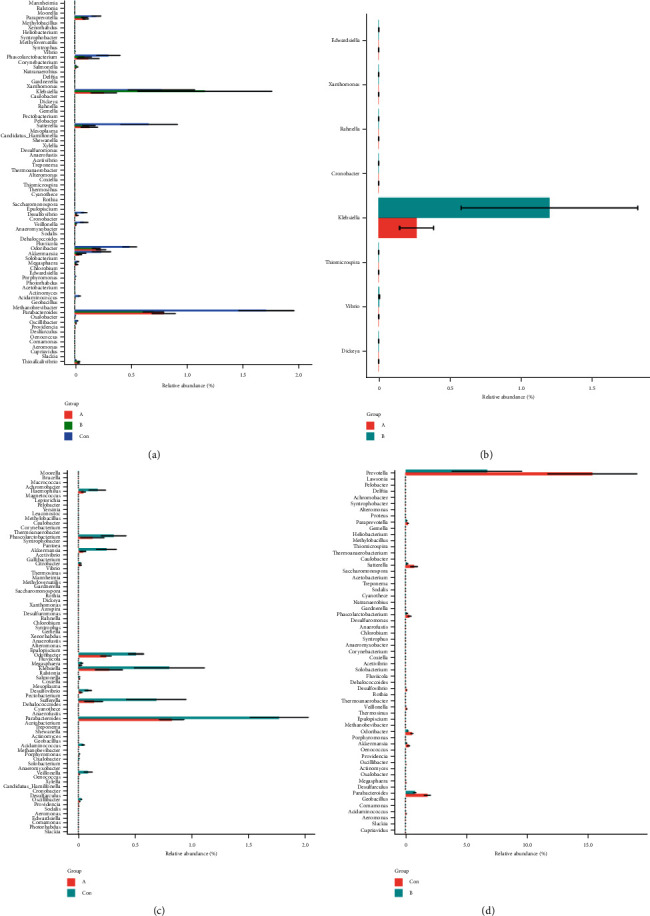
Difference species statistics between groups a-b-con at the genus level. (a–d) The group comparisons of a&b&con, a&b, a&con, and b&con, respectively.

Taking into account the influence of time accumulation, the change of gut microbiota required a process, and the interval between our treatments is about two weeks, which may affect the abundance of differential microbes. We compared the microbe species, gene, KO, and pathway abundance between different groups to detected the changes between the gout patients and healthy groups. By separately comparing the significantly different species between the two groups, we found that there were more significantly different species at the phylum level between group A vs Con than group B vs Con (Figure 2(a)). Among them, there was a significant difference in group A vs Con for *Proteobacteria*, but not significant in group B vs Con. Although *Proteobacteria* was not significant in group A vs B, its abundance had changed before and after treatment (Figure 2(a)). Although group A and group B did not have significantly different species at the phylum level, we detected some significantly different genera (Figure 2(b)). What we can clearly see was that these differences were concentrated in the *Proteobacteria* phylum, of which five genera were more in group A and less in group B.

**Figure 3:**
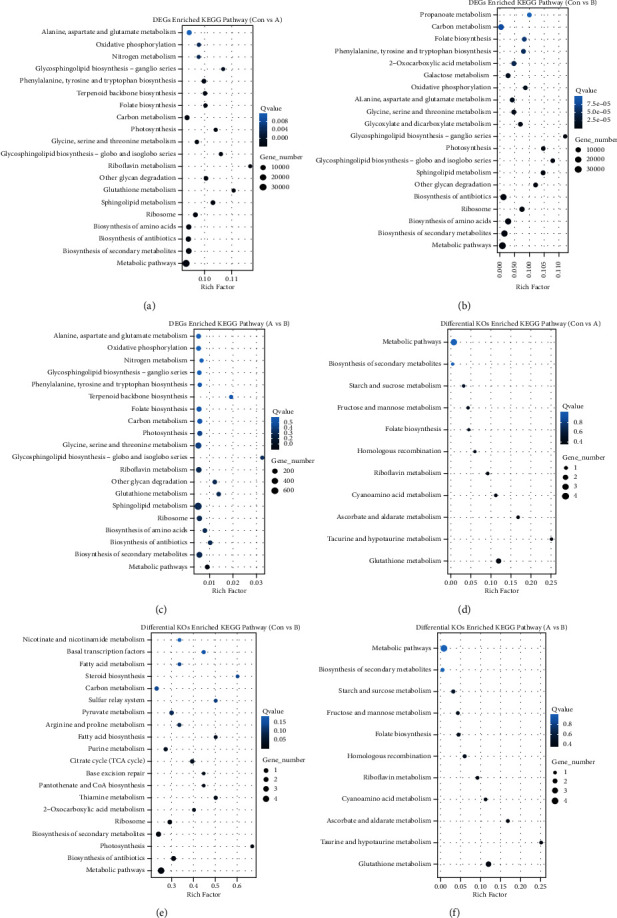
Bubble chart of KEGG pathway enrichment between each two groups. (a–c) The KEGG pathway comparison enriched in differentially expressed genes in two groups, respectively; (d–f) the KEGG pathway comparison enriched in differentially KOs in two groups, respectively.

Meanwhile, we calculated the difference of microbe gene abundance and KO abundance between the three groups, and then, the pathway enrichment analysis was carried out by using the differential genes or KOs. Based on differential expressed genes (DEGs) between each two groups, the group comparison DEG number of A vs Con or B vs Con was ten times than A vs B, which indicated that the huge microbe gene abundance difference between the gout patients and healthy groups (Figure 3 and Figure S2A–S2C). The pathways included glutathione metabolism, ubiquinone and other terpenoid-quinone biosynthesis, and tryptophan metabolism and pyruvate metabolism which were significantly enriched between group A vs B. Inconsistently, 45 pathways were significantly enriched between group A vs Con or group B vs Con, including metabolic pathways, biosynthesis of secondary metabolites, biosynthesis of antibiotics, and biosynthesis of amino acids (Figure 3). Meanwhile, only 153 differential KOs were detected between A-B and only the pathway, glutathione metabolism was significantly enriched, and biosynthesis of antibiotics, carbon metabolism, metabolic pathways, photosynthesis, and pyruvate metabolism were common significantly enriched between group A vs Con and group B vs Con (Figure 3 and Figures S2D-S2E). Moreover, there were 153 KEGG ontologies with significant differences between group A and B, included the previous significantly enriched pathways taurine and hypotaurine metabolism [27], folate biosynthesis [28], and fructose and mannose metabolism [29] (Figure 3).

**Figure 4:**
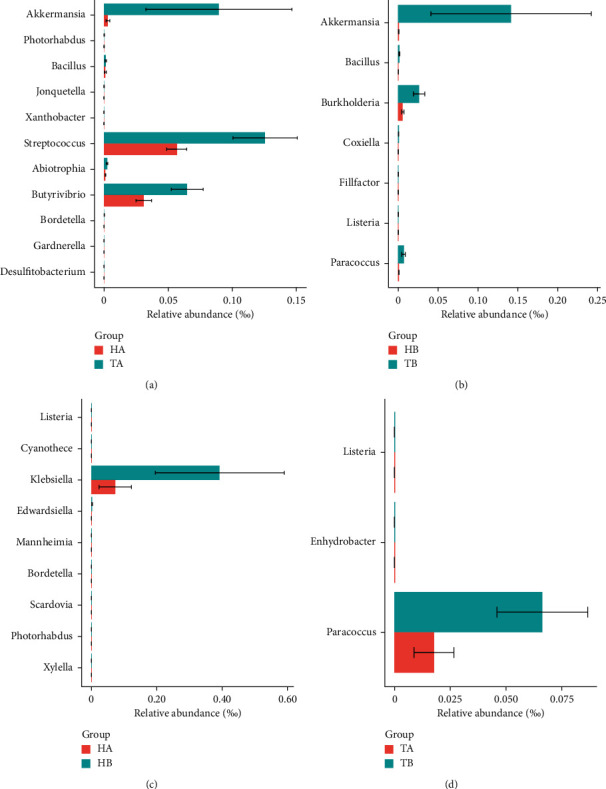
Difference species statistics between group H vs T at the genus level. (a) The changes of gut microbiota between group H vs T before medication; (b) the changes of gut microbiota between group H vs T after medication; (c) the changes of gut microbiota in group H before and after medication; (d) the changes of gut microbiota in group T before and after medication.

Furthermore, we also tried to find the different species between the H treatment group and the T treatment group after medication. The genus relative abundance result showed that the microbe composition between H treatment patients and T treatment patients were similar (Figure S1A), and before and after treatment in group T and H, we found no significant difference whether between TA and TB or between HA and HB (Figure S1B). For the different species detection, the abundance of *Verrucomicrobia* in the T group was significantly higher than that in the H group at the phylum level (Figure 4(a)). Moreover, at the genus level, *Butyrivibrio*, *Abiotrophia*, *Streptococcus*, and *Bacillus* belonging to *Firmicutes*, as well as *Akkermansia* belonging to Verrucomicrobia, were significantly more abundant in the T group than in the H group. There were *Desulfitobacterium* and *Photorhabdus* more in the H group than in the T group (Figure 4(b)). In terms of the number of genus with significant differences (*P* value ≤0.05), the T group was significantly more than the H group, which was exactly matched with the patient's phenotype, that the patients of the T group had more acute onset and more obvious inflammatory response than the patients of the H group. In order to exclude the interference of the age indicator, we also made a difference statistical analysis on all patients and all samples of control and found that there was no significant difference between the two groups showed by *t* test (*P*-value >0.05).

### 3.3. Changes Reflected in the B/F Ratio

The B : F ratio has been reported as an effective criterion for evaluating inflammation (Compare, Rocco, Sanduzzi [30]; M. [31]). Similarly, we used the B/F ratio to evaluate the impact on the composition and abundance of gut microbiota before and after treatment. The upregulation and downregulation expressions of six phyla in different individuals before and after medication, as well as the change of B :  F ratio, were counted (Table S3). After screening the samples with the same upregulation and downregulation, the two types were finally classified (Table S3).

**Figure 5:**
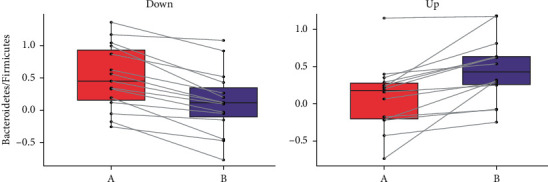
The B:F ratio and abundance (log2) in two groups. (a) The box plot of the change of B/F ratio in the down group for the patients before and after treatment. (b) The box plot of the change of B/F ratio in the up group for the patients before and after treatment. The change trend curves are given in each figure.

**Figure 6:**
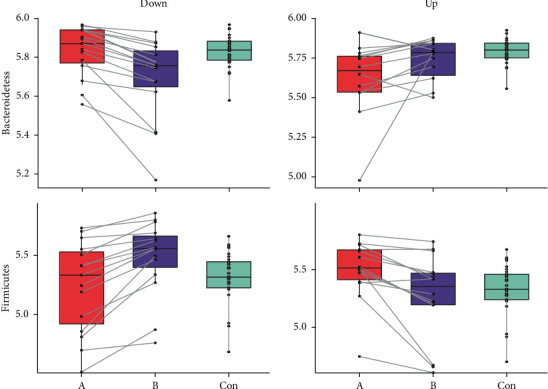
Trend chart of species abundance on the phylum level in three groups. (a) The box plot of the change of B/F ratio in the down group for three groups A&B&Con with respect to *Bacteroidetes* and *Firmicutes*. (b) The box plot of the change of B/F ratio in the up group for three groups A&B&Con with respect to *Bacteroidetes* and *Firmicutes*. The change trend curves are given in each figure.

By constructing the box plot of the B/F ratio of the down group and the up group, it was found that the trends of A and B in the down group were just opposite of those in the up group (Figure 5). The comparison of groups A-B-Con was performed on the down group and the up group, respectively, and it was found that the trend of *Bacteroidetes* and *Firmicutes* was relatively obvious in the down group. As for *Bacteroidetes*, it decreased from group A_down to group B_down and increased from group B_down to group Con (Figure 6). Regarding to *Firmicutes*, it had the opposite trend, which increased from group A_down to group B_down and decreased from group B_down to group Con (Figure 6). From these results in the down group, it can be found that the abundance of *Bacteroidetes* and *Firmicutes* in the patients before treatment and in the Con group appeared to be consistent and changed after treatment. However, in the up group, the abundance of *Bacteroidetes* and *Firmicutes* did not change significantly in the patients after treatment and in the Con group. Besides *Bacteroidetes* and Firmicutes, the trend of four other phyla including *Actinobacteria, Fusobacteria, Proteobacteria, and Verrucomicrobia* were performed (Figure S3).

### 3.4. Correlation Analysis between Medical Examination Index and Species

**Figure 7:**
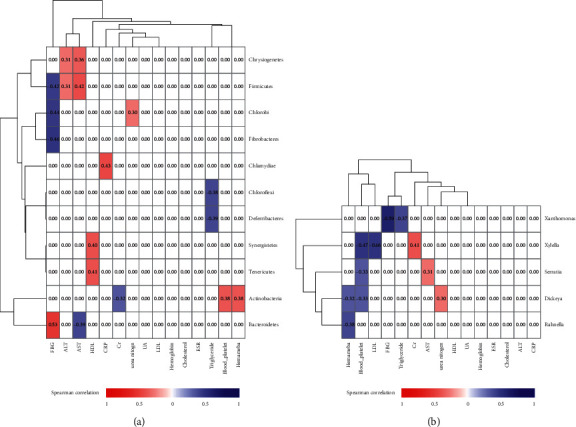
Correlation analysis between the medical examination index and species on the phylum level and genus level. (a) The correlation analysis between clinical test indicators and species at the phylum level; (b) the correlation analysis between clinical test indicators and species at the genus level. The *x* axis is the clinical test index; the *y* axis is the species name.

**Figure 8:**
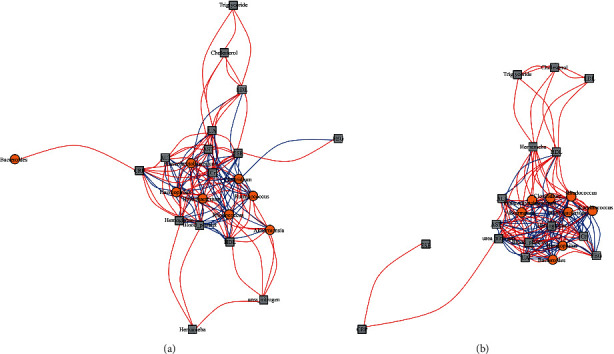
Interaction network diagram of primary focus species and clinical laboratory indicators between group a and b on the genus level. (a) The interaction diagram of focused species and clinical laboratory indicators by both a and b in the down group; (b) the interaction diagram of focused species and clinical laboratory indicators by both a and b in the up group.

Based on the obtained clinical laboratory indicators of 24 individuals (Table S4), correlation analysis was performed between the medical examination index and significantly different species (Figure 7), and also the interaction networks were constructed with significantly different species and clinical laboratory indicators between group A_down and B_down (Figure 8(a)) and group A_up and B_up (Figure 8(b)). From the interaction network diagram, some interesting associations were found; the association between species and clinical laboratory indicators in the up group was stronger than that in the down group, and *Bacteroides* had an obvious positive correlation with UA and Cr both of which were basically significantly correlated with the gout in the up group but had no correlation in the down group; seven other species which were *Akkermansia, Bifidobacterium, Clostridium, Haemophilus, Rhodococcus*, *Ruminococcus*, and *Phascolarctobacterium* had similar associations both in up and down groups (Figure 8). In addition, there were five species including *Bacteroides, Bifidobacterium, Clostridium, Haemophilus*, *and Phascolarctobacterium*, which had an obvious correlation with urea nitrogen in the up group but no correlation in the down group, and *Akkermansia, Rhodococcus* and *Ruminococcus* had similar associations both in up and down groups (Figure 8).

## 4. Discussion

TCM can benefit patients through treatment based on syndrome differentiation, which is the unique advantage of Chinese medicine. According to the theory of TCM, “dampness” and “heat” are the causative factors of gouty arthritis, which is also associated with congenital deficiency and dysfunction of the spleen and kidney. In the acute attack phase, the syndrome of dampness-heat toxin amassment and dampness-heat with blood stasis are the commonest diagnostic type. Tongfengtai is an effective herbal formula used for treating gouty arthritis through clearing away heat, eliminating dampness, and detoxifying, which mainly consisted of heat-clearing and detoxifying herbs.

Pathogenesis and syndrome type transformation is another vital principle based on the TCM theory, the disease for a long time will be deficient and create a new way of thinking for influence the spleen and kidney, or change to blood stasis. It refers to the pathological state that manifests in the development and change process of the evil and the positive as both excess syndrome and deficiency syndrome in clinical. It will be transformed to the damp-heat combination of spleen or kidney deficiency syndrome, or phlegm and blood stasis obstruction syndrome. Hushen Tongfengtai is recommended to be used for restraining gouty arthritis through dispelling dampness, clearing away heat in addition to invigorating the spleen and kidney, which mainly consisted of invigorating medicine besides heat-clearing and dampness-dispelling herbs.

In the abovementioned results, we knew that the significantly enriched pathway of genes between groups A and B was glutathione metabolism. Glutamate involved in the glutathione metabolism was not only a major metabolite in the Krebs cycle and a precursor for uric acid synthesis but also inhibited the glutamate-cystine antitransport system, resulting in a significant reduction in intracellular glutathione levels. The pathway ID ko00480 of the glutathione metabolism had an increase in abundance from before treatment to after treatment. K00799, as the gene related with the glutathione metabolism, the mean of that in group A and group B were 0.005803 and 0.011429, respectively, which can be speculated on the correlation of the glutathione metabolism before and after treatment in gout patients.

The known clinical laboratory indicators closely related to gout were UA, Cr, and urea nitrogen, we have found the association trend of different species and clinical laboratory indicators in the up and down groups. One of the conclusions was that, *Akkermansia* reported to be closely related to gout which was correlated with three major indicators both in the up and down groups [20,32], of which UA and Cr were negatively correlated, and urea nitrogen was positively correlated. As known from previous studies, the abundance and diversity of *Bacteroides* varied significantly between individuals [19], and our conclusion was also consistent with that. Specifically, *Bacteroides* was correlated with key indicators in patients in the up group but not in patients in the down group.

From the significantly different species at the genus level between group A and group B, we found these genera mainly belonged to the *Proteobacteria* phylum, of which five genera were more in group A and less in group B, and they were all pathogenic bacteria, so it can be easily understood that our drugs help inhibiting the reproduction of pathogenic bacteria of these five genera and help patients to restore normal intestinal levels. However, there was also a strange phenomenon that *Klebsiella* increased in the intestines of patients after treatment with drugs, whether it was because of the effect of the drug or because of the decreased abundance of other bacteria, needs to be further studied.

## 5. Conclusion

The present study performed metagenomic analysis of gut microbiome in gout patients with different Chinese traditional medicine treatment (T treatment and H treatment). After the treatment, the glutathione pathway was significantly enriched and some pathogenic bacteria were significantly inhibited. In conclusion, the present study suggests that traditional Chinese medicine treatment may exert its therapeutic effect by inhibiting relevant pathways.

## Figures and Tables

**Table 1:** Sample number and group information.

Group	Treatment	Human no.	Taking medicine	Group name	Sample no.
Patient	H	16	Before	HA	16
			After	HB	16
	T	14	Before	TA	14
			After	TB	14
Control	—	29	—	Con	29

## Data Availability

All data used during the study are available from the corresponding author on reasonable request.

## Supplementary Materials

Supplementary Materials The present study contains 7 supplemental material files, including 3 supplementary figures and 4 supplementary tables; the legends are as followed: (1) *Figure S1*. Genus abundance and PCoA analysis for four treatment groups (please refer to Figure S1.docx). (2) *Figure S2*. Differential expressed gene and KOs pathway enrichment (please refer to Figure S2.docx). (3) *Figure S3*. Trend chart of other species abundance on phylum level in three groups (please refer to Figure S3.docx). (4) *Table S1*. Sequencing data summary (please refer to Table S1.pdf). (5) *Table S2.* Phylum annotation and relative abundance in three groups (please refer to Supplementary data.docx). (6) *Table S3.* Statistics of selected upregulation and downregulation samples (please refer to Supplementary data.docx). (7) *Table S4*. Biochemical indicators (please refer to Table S4.pdf).

## References

[1] Scuiller A., Pascart T., Bernard A., Oehler E. (2020). Gout. *La Revue de Medecine Interne*.

[2] Jamnik J., Rehman S., Mejia S. B. (2016). Fructose intake and risk of gout and hyperuricemia: a systematic review and meta-analysis of prospective cohort studies. *BMJ Open*.

[3] Li R., Yu K., Li C. (2018). Dietary factors and risk of gout and hyperuricemia: a meta-analysis and systematic review. *Asia Pacific Journal of Clinical Nutrition*.

[4] Dalbeth N., Merriman T. R., Stamp L. K. (2016). Gout. *The Lancet*.

[5] Grassi W., De Angelis R. (2012). Clinical features of gout. *Reumatismo*.

[6] Becker M. A., Schumacher H. R., Wortmann R. L. (2005). Febuxostat compared with allopurinol in patients with hyperuricemia and gout. *New England Journal of Medicine*.

[7] White W. B., Saag K. G., Becker M. A. (2018). Cardiovascular safety of Febuxostat or allopurinol in patients with gout. *New England Journal of Medicine*.

[8] Li W. H., Han J. R., Ren P. P., Xie Y., Jiang D. Y. (2021). Exploration of the mechanism of Zisheng Shenqi decoction against gout arthritis using network pharmacology. *Computational Biology and Chemistry*.

[9] Liu P., Xu H., Shi Y., Deng L., Chen X. (2020). Potential molecular mechanisms of plantain in the treatment of gout and hyperuricemia based on network pharmacology. *Evidence-based Complementary and Alternative Medicine*.

[10] Chen J., Wu M., Yang J., Wang J., Qiao Y., Li X. (2017). The immunological basis in the pathogenesis of gout. *Iranian Journal Immunology*.

[11] Kawamura Y., Nakaoka H., Nakayama A. (2019). Genome-wide association study revealed novel loci which aggravate asymptomatic hyperuricaemia into gout. *Annals of the Rheumatic Diseases*.

[12] Tseng C. C., Wong M. C., Liao W. T. (2020). Systemic investigation of promoter-wide methylome and genome variations in gout. *International Journal of Molecular Sciences*.

[13] Qin J., Li Y., Cai Z. (2012). A metagenome-wide association study of gut microbiota in type 2 diabetes. *Nature*.

[14] Pernigoni N., Zagato E., Calcinotto A. (2021). Commensal bacteria promote endocrine resistance in prostate cancer through androgen biosynthesis. *Science*.

[15] Nikolova V. L., Hall M. R. B., Hall L. J., Cleare A. J., Stone J. M., Young A. H. (2021). Perturbations in gut microbiota composition in psychiatric disorders: a review and meta-analysis. *JAMA Psychiatry*.

[16] Chen J., Wright K., Davis J. M. (2016). An expansion of rare lineage intestinal microbes characterizes rheumatoid arthritis. *Genome Medicine*.

[17] Marietta E. V., Murray J. A., Luckey D. H. (2016). Suppression of inflammatory arthritis by human gut-derived prevotella histicola in humanized mice. *Arthritis & Rheumatology*.

[18] Maiuolo J., Oppedisano F., Gratteri S., Muscoli C., Mollace V. (2016). Regulation of uric acid metabolism and excretion. *International Journal of Cardiology*.

[19] Xing S. C., Meng D. M., Chen Y. (2015). Study on the diversity of Bacteroides and Clostridium in patients with primary gout. *Cell Biochemistry and Biophysics*.

[20] Mendez-Salazar E. O., Vazquez-Mellado J., Casimiro-Soriguer C. S. (2021). Taxonomic variations in the gut microbiome of gout patients with and without tophi might have a functional impact on urate metabolism. *Molecular Medicine*.

[21] Shao T., Shao L., Li H., Xie Z., He Z., Wen C. (2017). Combined signature of the fecal microbiome and metabolome in patients with gout. *Frontiers in Microbiology*.

[22] Guo Z., Zhang J., Wang Z. (2016). Intestinal microbiota distinguish gout patients from healthy humans. *Scientific Reports*.

[23] Langmead B., Trapnell C., Pop M., Salzberg S. L. (2009). Ultrafast and memory-efficient alignment of short DNA sequences to the human genome. *Genome Biology*.

[24] Li J., Jia H., Cai X. (2014). An integrated catalog of reference genes in the human gut microbiome. *Nature Biotechnology*.

[25] Patro R., Duggal G., Love M. I., Irizarry R. A., Kingsford C. (2017). Salmon provides fast and bias-aware quantification of transcript expression. *Nature Methods*.

[26] Matsouaka R. A., Singhal A. B., Betensky R. A. (2018). An optimal Wilcoxon-Mann-Whitney test of mortality and a continuous outcome. *Statistical Methods in Medical Research*.

[27] Huang B., Hu X., Wang J., Li P., Chen J. (2019). Study on chemical constituents of herbal formula Er Miao Wan and GC-MS based metabolomics approach to evaluate its therapeutic effects on hyperuricemic rats. *Journal of Chromatography B*.

[28] Itou S., Goto Y., Suzuki K. (2009). Significant association between methylenetetrahydrofolate reductase 677T allele and hyperuricemia among adult Japanese subjects. *Nutrition Research*.

[29] Chu Y., Sun S., Huang Y. (2021). Metagenomic analysis revealed the potential role of gut microbiome in gout. *NPJ Biofilms Microbiomes*.

[30] Compare D., Rocco A., Zamparelli M. S., Nardone G. (2016). The gut bacteria-driven obesity development. *Digestive Diseases*.

[31] Liu M., Ma L., Chen Q. (2018). Fucoidan alleviates dyslipidemia and modulates gut microbiota in high-fat diet-induced mice. *Journal of Functional Foods*.

[32] Xi Y., Huang Y., Li Y., Yan J., Shi Z. (2020). Fermented feed supplement relieves caecal microbiota dysbiosis and kidney injury caused by high-protein diet in the development of gosling gout. *Animals*.

