# Supplementary material for: Metagenomic Analysis of Gut Microbiome in Gout Patients with Different Chinese Traditional Medicine Treatments
Source: Evid Based Complement Alternat Med. 2022 Oct 10;2022:6466149. doi: 10.1155/2022/6466149 (PMC9576389; doi:10.1155/2022/6466149)
Supplement: Supplementary Materials — The present study contains 7 supplemental material files, including 3 supplementary figures and 4 supplementary tables; the legends are as followed: (1) Figure S1. Genus abundance and PCoA analysis for four treatment groups (please refer to Figure S1.docx). (2) Figure S2. Differential expressed gene and KOs pathway enrichment (please refer to Figure S2.docx). (3) Figure S3. Trend chart of other species abundance on phylum level in three groups (please refer to Figure S3.docx). (4) Table S1. Sequencing data summary (please refer to Table S1.pdf). (5) Table S2. Phylum annotation and relative abundance in three groups (please refer to Supplementary data.docx). (6) Table S3. Statistics of selected upregulation and downregulation samples (please refer to Supplementary data.docx). (7) Table S4. Biochemical indicators (please refer to Table S4.pdf). [file 6466149.f1.zip › 6466149.f1/Supplementary data (2).docx]

**Supplemental Figure and Table Legends**

**
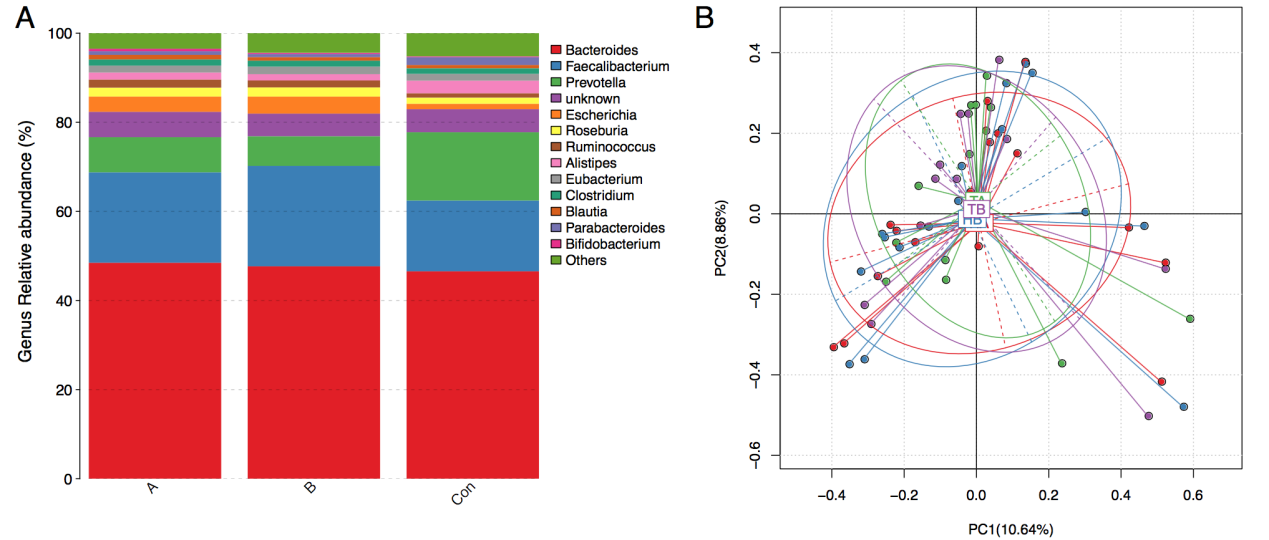
**

**Figure S1. Genus abundance and PCoA analysis for four treatment groups.**

**
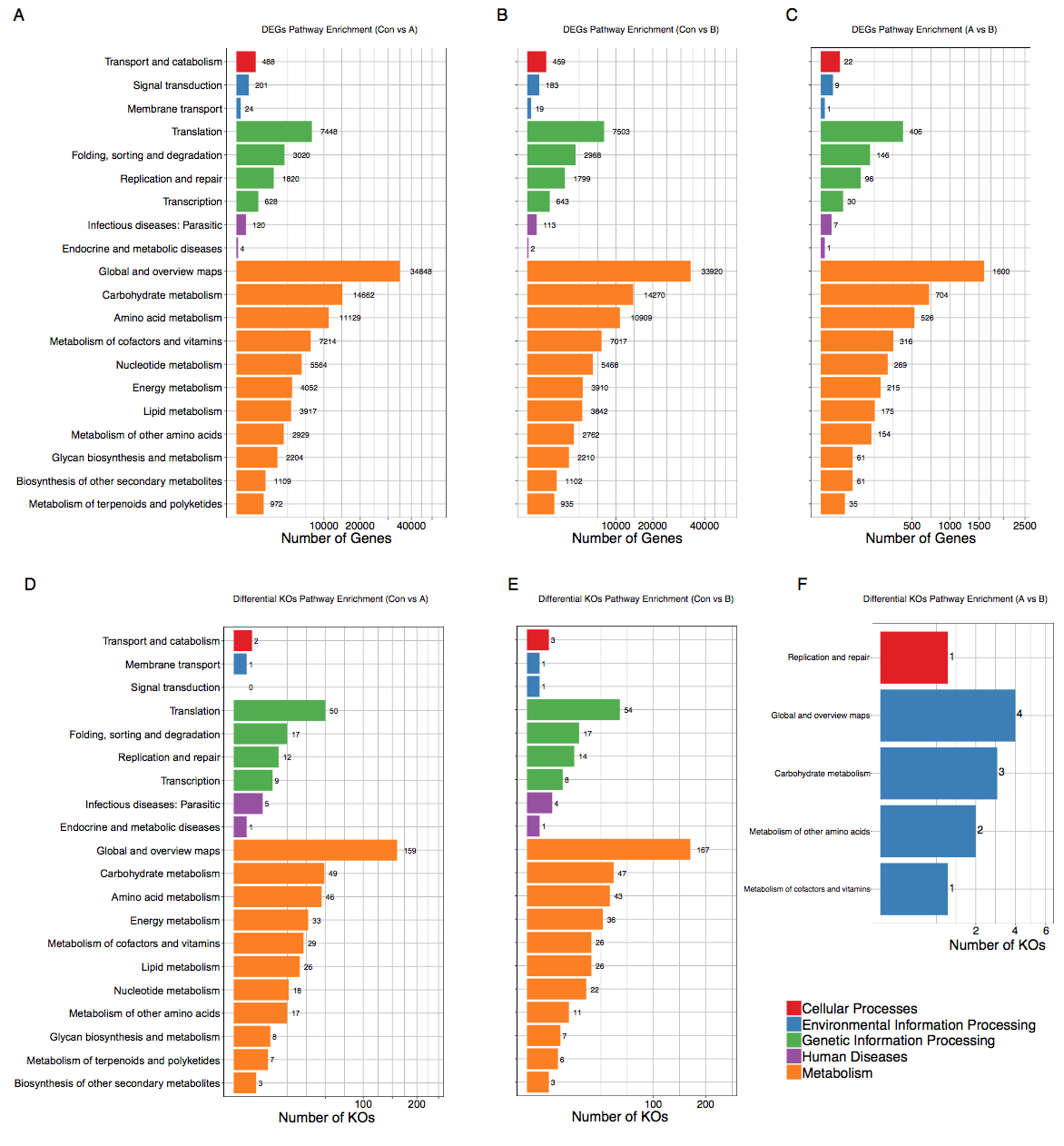
**

**Figure S2. Differential expressed gene and KOs pathway enrichment.**

**
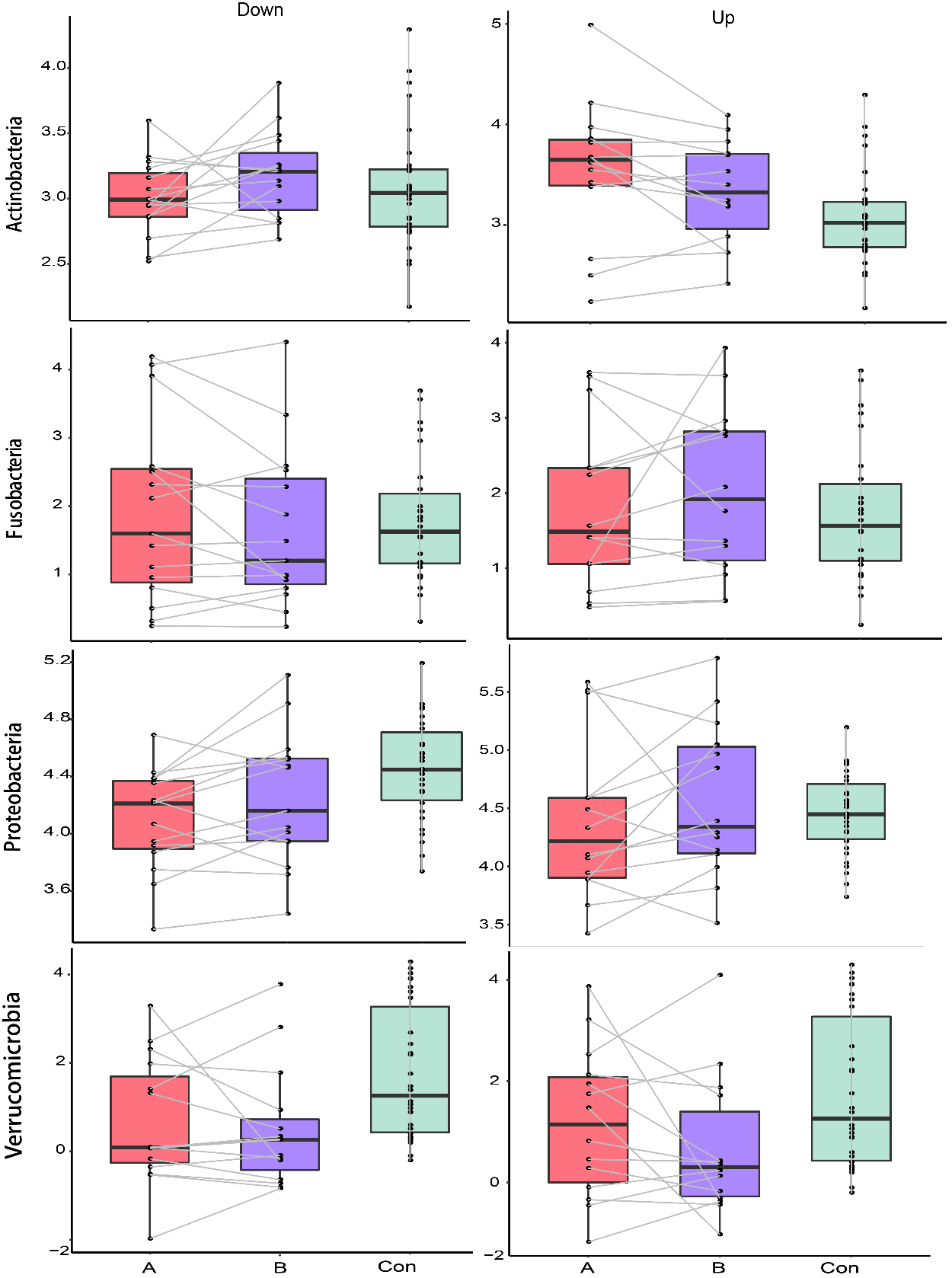
**

**Figure S3. Trend chart of other species abundance on phylum level in three groups.**

**Table S1. Sequencing data summary.**

Please refer to TableS1.xls.

**Table S2. Phylum annotation and relative abundance in three groups.**

| **Phylum** | **Gene Number** | **Gene rate(%)** | **Abundance**  **(group A)** | **Abundance**  **(group A)** | **Abundance**  **(gourp Con)** |
| --- | --- | --- | --- | --- | --- |
| Bacteroidetes | 434,380 | 25.24 | 60.51119 | 57.894274 | 69.112055 |
| Firmicutes | 937,605 | 54.48 | 33.733844 | 35.215955 | 26.452701 |
| Proteobacteria | 211,483 | 12.29 | 4.901471 | 6.311783 | 3.874871 |
| Actinobacteria | 92,961 | 5.40 | 0.62147 | 0.325004 | 0.237825 |
| Fusobacteria | 19,158 | 1.11 | 0.182252 | 0.178016 | 0.052395 |
| Verrucomicrobia | 11,429 | 0.66 | 0.041167 | 0.064986 | 0.253617 |
| Other | 13,864 | 0.80 | 0.008605 | 0.009981 | 0.016538 |

**Table S3. Statistics of selected up-regulation and down-regulation samples.**

|  | **Bacteroidetes** | **Firmicutes** | **Proteobacteria** | **Actinobacteria** | **Fusobacteria** | **Verrucomicrobia** | **Bacteroidetes** |
| --- | --- | --- | --- | --- | --- | --- | --- |
|  |  |  |  |  |  |  | **/Firmicutes** |
| 101(A-B) | down | up | up | up | up | down | down |
| 102(A-B) | down | up | up | down | down | up | down |
| 103(A-B) | down | up | up | up | down | up | down |
| 104(A-B) | down | up | up | up | down | down | down |
| 107(A-B) | down | up | down | up | down | down | down |
| 108(A-B) | down | up | down | up | down | up | down |
| 113(A-B) | down | up | up | up | up | down | down |
| 114(A-B) | down | up | up | up | up | down | down |
| 303(A-B) | down | up | down | down | up | up | down |
| 311(A-B) | down | up | up | up | down | up | down |
| 402(A-B) | down | up | down | down | up | up | down |
| 403(A-B) | down | up | up | up | down | down | down |
| 407(A-B) | down | up | up | up | up | up | down |
| 409(A-B) | down | up | up | up | up | down | down |
| 410(A-B) | down | up | up | down | down | down | down |
| 109(A-B) | up | down | up | up | up | down | up |
| 110(A-B) | up | down | up | up | down | down | up |
| 112(A-B) | up | up | down | up | down | up | up |
| 115(A-B) | up | down | up | up | up | up | up |
| 201(A-B) | up | down | up | down | up | down | up |
| 301(A-B) | down | down | up | up | down | down | up |
| 302(A-B) | up | down | up | down | up | down | up |
| 306(A-B) | up | down | up | down | up | up | up |
| 307(A-B) | up | down | up | down | down | up | up |
| 308(A-B) | up | up | down | down | down | up | up |
| 404(A-B) | up | down | down | down | up | down | up |
| 405(A-B) | down | down | up | up | up | down | up |
| 406(A-B) | up | down | up | down | up | down | up |
| 408(A-B) | up | down | up | down | up | down | up |
| 411(A-B) | up | down | down | up | up | up | up |
| Total | up13 down17 | up17 down13 | up22 down8 | up18 down12 | up17 down13 | up13 down17 | up15 down15 |

**Table S4. Biochemical indicators.**

Please refer to TableS4.xls.
