# Supplementary material for: Metagenomic Analysis of Gut Microbiome in Gout Patients with Different Chinese Traditional Medicine Treatments
Source: Evid Based Complement Alternat Med. 2022 Oct 10;2022:6466149. doi: 10.1155/2022/6466149 (PMC9576389; doi:10.1155/2022/6466149)
Supplement: Supplementary Materials — The present study contains 7 supplemental material files, including 3 supplementary figures and 4 supplementary tables; the legends are as followed: (1) Figure S1. Genus abundance and PCoA analysis for four treatment groups (please refer to Figure S1.docx). (2) Figure S2. Differential expressed gene and KOs pathway enrichment (please refer to Figure S2.docx). (3) Figure S3. Trend chart of other species abundance on phylum level in three groups (please refer to Figure S3.docx). (4) Table S1. Sequencing data summary (please refer to Table S1.pdf). (5) Table S2. Phylum annotation and relative abundance in three groups (please refer to Supplementary data.docx). (6) Table S3. Statistics of selected upregulation and downregulation samples (please refer to Supplementary data.docx). (7) Table S4. Biochemical indicators (please refer to Table S4.pdf). [file 6466149.f1.zip › 6466149.f1/TableS1 (1).pdf]

Sample_Name	w_Reads	ean_Reads (	ean_Bases (G	ndata_Ratio	ost_Clean_Base	n_Reads Q20	n_Reads_Q30	Mapped_reads	apped_rate(%	ped_IGC_gen	Age	Gender	Clinical Type	Years of onset	Treatment	Situation	Hemameba	Hemoglobin	Blood_platele	ALT	AST	UA	Cr	urea nitrogen	FBG	Triglyceride	HDL	LDL	Cholesterol	ESR	CRP
101A	167.77	149.17	14.91	88.91	14.91	97.75	93.11	119,706,945	80.28	658,209	33	Male	mbination of spleen or kidney deficie	7 Years	Hushentongfengtai	Before treatment				40.9	29.9	558	67	3.6	5.09	5.24	0.71	2.74	4.81		
101B	179.25	158.40	15.83	88.37	15.83	97.65	92.80	122,181,337	77.37	639,413	33	Male	mbination of spleen or kidney deficie	7 Years	Hushentongfengtai	After treatment	8.19	155	394	30.6	24.7	394	79	4.6	4.58	2.49	0.82	3.96	5.6	2	1
102A	204.00	183.89	18.39	90.14	18.39	97.87	93.25	147,357,040	80.16	593,438	47	Male	dampness-heat with blood stasis	10 Years	Tongfengtai	Before treatment	12.3	130	385	18.6	12.3		90	5.1	6.34					41	53.6
102B	154.25	135.59	13.55	87.90	13.55	97.68	92.76	106,751,257	78.80	654,747	47	Male	dampness-heat with blood stasis	10 Years	Tongfengtai	After treatment	5.72	162	260	52.3	31.6	346	87	4.8	5.07	2.81	0.92	4.25	5.79	2	2.8
103A	189.05	167.69	16.75	88.70	16.75	97.69	92.95	127,303,425	75.97	702,444	45	Male	dampness-heat with blood stasis	2 Years	Tongfengtai	Before treatment						487	86	2.4	5.01	1.37	1.4	3.81	5.36		
103B	191.10	169.58	16.95	88.74	16.95	97.70	92.96	132,997,165	78.99	775,000	45	Male	dampness-heat with blood stasis	2 Years	Tongfengtai	After treatment						599	98	3.3		1.08	1.5	3.86	5.38		
104A	217.10	197.33	19.71	90.89	19.71	97.77	93.18	160,044,859	81.13	854,375	38	Male	Phlegm and blood stasis obstruction	8 Years	Hushentongfengtai	Before treatment	19.6	100	467												
104B	211.85	194.39	19.42	91.76	19.42	97.81	93.34	157,437,992	81.02	963,151	38	Male	Phlegm and blood stasis obstruction	8 Years	Hushentongfengtai	After treatment	6.98	121	380	17.9	17.1	695	95	55	4.96	2.77	0.92	2.96	4.85	35	15.3
107A	151.57	134.68	13.46	88.86	13.46	97.62	92.34	107,763,564	80.15	510,184	39	Male	mbination of spleen or kidney deficie	4 Years	Hushentongfengtai	Before treatment				16.9	20	378	113	4.6							
107B	179.94	161.26	16.12	89.62	16.12	97.71	92.58	131,897,082	81.82	591,894	39	Male	mbination of spleen or kidney deficie	4 Years	Hushentongfengtai	After treatment	7.73	161	229	26	21.6	435	110	4.3	5.42	2.04			5.44	6	2.6
108A	141.32	126.36	12.63	89.41	12.63	97.69	92.57	100,056,617	79.22	746,269	32	Male	Phlegm and blood stasis obstruction	2 Years	Hushentongfengtai	Before treatment	8.98	164	315	34.7	24.5	600	81	4	4.95	1.81			5.2	2	1.5
108B	119.87	107.33	10.73	89.54	10.73	97.77	92.95	85,321,230	79.57	746,727	32	Male	Phlegm and blood stasis obstruction	2 Years	Hushentongfengtai	After treatment	7.26	151	337	15.9	16.8	405	85	3.7		1.32	0.85	2.93	4.15	11	16.3
109A	125.66	107.40	10.73	85.47	10.73	97.03	91.75	82,655,064	77.00	890,200	56	Male	Phlegm and blood stasis obstruction	2 Years	Hushentongfengtai	Before treatment	7	139	272	12.5	22.5	180	93	6.7						51	5.1
109B	159.33	138.38	13.84	86.85	13.84	97.11	91.62	109,888,163	79.44	1,079,581	56	Male	Phlegm and blood stasis obstruction	2 Years	Hushentongfengtai	After treatment				27	30.7	390	97	5						16	2.3
110A	134.05	119.69	11.91	89.29	11.91	97.50	93.02	77,962,194	65.58	755,771	25	Male	Phlegm and blood stasis obstruction	1 Year	Hushentongfengtai	Before treatment															
110B	178.21	156.73	15.66	87.95	15.66	97.35	92.34	127,376,713	81.34	636,964	25	Male	Phlegm and blood stasis obstruction	1 Year	Hushentongfengtai	After treatment															
112A	226.11	201.16	20.11	88.97	20.11	97.39	92.37	160,194,272	79.72	844,647	58	Male	Phlegm and blood stasis obstruction	13 Years	Hushentongfengtai	Before treatment	5.95	159	286	89.4	64.1	616	103	5.3	5.21	1.61	0.94	2.86	4.34	15	11
112B	158.15	137.65	13.76	87.04	13.76	97.22	92.00	109,971,093	80.03	821,819	58	Male	Phlegm and blood stasis obstruction	13 Years	Hushentongfengtai	After treatment	5.5	159	249	36	57.8	404	102	4.1		1.82	0.88	3.8	5.16	8	2.1
113A	165.97	144.80	14.48	87.24	14.48	97.32	92.43	114,744,600	79.27	813,497	51	Male	Phlegm and blood stasis obstruction	13 Years	Hushentongfengtai	Before treatment	5.87	163	254	23.2	21.4	461	120	5	6.07						
113B	154.30	134.14	13.41	86.93	13.41	97.26	92.26	104,643,868	78.22	870,303	51	Male	Phlegm and blood stasis obstruction	13 Years	Hushentongfengtai	After treatment	4.89	164	213	19.1	17.3	415	113	4	6.53						
114A	174.19	151.70	15.17	87.09	15.17	97.29	92.26	122,052,544	80.69	923,336	49	Male	dampness-heat toxin amassment	7 Years	Tongfengtai	Before treatment	4.69	133	74	22.9	19.8	238	129	4.9	4.89	0.81	1.48	1.78	3.46	18	61.2
114B	174.00	151.36	15.13	86.99	15.13	97.31	92.35	119,841,060	79.65	800,459	49	Male	dampness-heat toxin amassment	7 Years	Tongfengtai	After treatment	4.87	142	89	28.1	27.1	752	114	3.4							
115A	212.04	187.69	18.75	88.52	18.75	97.44	92.76	152,416,531	81.95	668,768	24	Male	dampness-heat toxin amassment	1 Year	Tongfengtai	Before treatment															
115B	151.02	132.13	13.21	87.49	13.21	97.39	92.64	105,025,856	79.62	533,663	24	Male	dampness-heat toxin amassment	1 Year	Tongfengtai	After treatment															
201A	239.07	220.48	21.99	92.22	21.99	97.94	93.44	174,414,082	79.14	921,625	44	Male	dampness-heat with blood stasis	7 Years	Tongfengtai	Before treatment	6.1	148	298	28.9	26.7	248	86	4.5		1	1.19	3.6	5.23		
201B	165.98	148.47	14.83	89.45	14.83	97.79	92.80	113,293,277	76.37	774,469	44	Male	dampness-heat with blood stasis	7 Years	Tongfengtai	After treatment	7.46	153	315	19.2	21.9	568	83	3.9	5.02	1.15	1.14	4.14	5.7	5	0.3
301A	144.96	134.53	13.45	92.80	13.45	98.14	93.47	109,315,062	81.78	525,612	54	Male	Phlegm and blood stasis obstruction	9 Years	Hushentongfengtai	Before treatment	8.05	149	294	14.3	13.5	572	87	5.8	5.04	2.27	1.03	5.23	6.6	3	1.1
301B	220.12	204.86	20.47	93.07	20.47	98.15	93.68	168,063,172	82.09	573,900	54	Male	Phlegm and blood stasis obstruction	9 Years	Hushentongfengtai	After treatment	9.9	6.29	300	13.6	11.7	283	83	6.1						9	17.8
302A	182.99	168.18	16.81	91.91	16.81	97.84	92.86	137,415,848	81.87	593,901	64	Male	dampness-heat with blood stasis	3 Years	Tongfengtai	Before treatment	8.71	122	419	11.2	15.7	262	53	4.3	6.67	3.66	1.23	3.35	5.66	70	10.8
302B	176.77	162.47	16.24	91.91	16.23	97.90	93.00	127,035,552	80.89	414,100	64	Male	dampness-heat with blood stasis	3 Years	Tongfengtai	After treatment	8.38	120	395	12.3	13.5	587	69	5.4	7.63	1.89	1.29	2.58	4.29		
303A	168.84	155.98	15.59	92.38	15.59	97.94	93.36	122,215,155	79.01	761,000	56	Male	dampness-heat with blood stasis	1 Year	Tongfengtai	Before treatment	4.07	146	155	18.2	27.3	426	85	2.8	5.19	2.87	0.82	2.3	3.96	14	1
303B	143.76	132.46	13.24	92.14	13.24	97.88	93.07	104,427,408	78.89	832,329	56	Male	dampness-heat with blood stasis	1 Year	Tongfengtai	After treatment	4.18	151	141	15.4	17.7	513	89	4		3.05	0.971	2.44	4.23		
306A	182.96	168.40	16.84	92.04	16.84	97.91	92.91	136,976,270	81.48	668,408	41	Male	dampness-heat toxin amassment	11 Years	Tongfengtai	Before treatment	7.48	162	259	64.9	36.9	303	79	2.4	5.52	4.31	0.88	3.29	5.38	1	0.9
306B	189.45	174.56	17.45	92.14	17.45	97.93	92.99	142,084,257	81.45	801,968	41	Male	dampness-heat toxin amassment	11 Years	Tongfengtai	After treatment	7.95	163	288	73.9	39.6	319	85	2.8	6.16	2.93	0.96	4.06	5.59	1	1.1
307A	201.78	179.64	17.94	89.03	17.94	97.27	92.53	145,502,357	81.30	548,195	53	Male	dampness-heat toxin amassment	27 Years	Tongfengtai	Before treatment	7.09	171	241	26.4	21.5	655	99	3.5	5.52	1.92	0.96	4.32	5.73	11	7.4
307B	188.75	162.86	16.28	86.28	16.28	96.96	91.58	132,652,318	81.80	574,728	53	Male	dampness-heat toxin amassment	27 Years	Tongfengtai	After treatment	6.32	168	197	26.8	21.7	498	97	3.3	6.55	2.19	1.1	3.9	5.72		
308A	177.26	151.47	15.14	85.45	15.14	96.91	91.62	117,033,214	77.39	577,637	63	Male	dampness-heat with blood stasis	10 Years	Tongfengtai	Before treatment	7.85	137	351	17.4	18.9	491	76	4.6		4.61	1.2	4.97	6.72	10	5.3
308B	138.90	118.97	11.89	85.65	11.89	96.93	91.76	93,582,797	80.04	506,012	63	Male	dampness-heat with blood stasis	10 Years	Tongfengtai	After treatment	10.24	132	396	12.6	17.8	351	80	4.7		1.88	1.31	3.09	5.03	27	16.2
311A	164.90	143.14	14.31	86.80	14.31	97.01	91.72	113,957,499	79.82	540,916	47	Male	dampness-heat with blood stasis	12 Years	Tongfengtai	Before treatment	4.96	5.11	224	10.8	16.6	328	94	3.8	6.15	4.7	0.81	3.43	5.92	7	1.9
311B	152.01	131.08	13.11	86.23	13.10	97.00	91.77	101,561,645	80.68	742,687	47	Male	dampness-heat with blood stasis	12 Years	Tongfengtai	After treatment	6.25	136	158	5.6	10.8	285	86	3.2							
402A	69.12	57.83	5.78	83.67	5.78	96.95	91.53	43,331,422	75.07	356,146	41	Male	Phlegm and blood stasis obstruction	1 Year	Hushentongfengtai	Before treatment				20.7	17.8	393	93	3.6							
402B	104.95	87.65	8.76	83.52	8.76	96.76	91.10	67,784,261	77.54	430,329	41	Male	Phlegm and blood stasis obstruction	1 Year	Hushentongfengtai	After treatment						522	86	2.5							
403A	191.40	166.33	16.63	86.90	16.63	97.07	91.79	134,234,421	80.77	710,758	45	Male	dampness-heat with blood stasis	8 Years	Tongfengtai	Before treatment				28	19.7	431	84	5.1							
403B	194.49	168.61	16.86	86.69	16.86	97.06	91.90	135,694,909	80.74	490,137	45	Male	dampness-heat with blood stasis	8 Years	Tongfengtai	After treatment	11.34	156	250	44	24	577	85	4.4		3.63	0.7	2.76	4.05		
404A	125.79	105.63	10.56	83.97	10.56	96.81	91.41	80,199,334	76.98	335,637	57	Male	Phlegm and blood stasis obstruction	5 Years	Hushentongfengtai	Before treatment	6.55	159	199	29.7	26	536	76	2.2		2.17			4.19	2	0.8
404B	226.83	199.07	19.90	87.76	19.90	97.17	92.18	162,880,758	81.90	426,874	57	Male	Phlegm and blood stasis obstruction	5 Years	Hushentongfengtai	After treatment				36.4	29.7	269	75	3.1							
405A	199.41	182.54	18.25	91.54	18.25	97.89	93.16	145,029,495	79.58	660,378	27	Male	dampness-heat with blood stasis	2 Years	Tongfengtai	Before treatment															
405B	153.26	138.60	13.86	90.43	13.86	97.75	92.65	107,280,174	77.82	318,781	27	Male	dampness-heat with blood stasis	2 Years	Tongfengtai	After treatment															
406A	165.32	153.03	15.30	92.57	15.30	97.97	93.32	123,060,002	80.43	904,177	24	Male	Phlegm and blood stasis obstruction	3 Years	Hushentongfengtai	Before treatment	6.05	170	242	31.7	23.1	740	108	4.4	4.64	2.82	0.99	3.73	5.52	4	0.8
406B	214.59	197.78	19.78	92.17	19.77	98.07	93.65	160,780,264	81.32	902,536	24	Male	Phlegm and blood stasis obstruction	3 Years	Hushentongfengtai	After treatment	6.15	157	232	32.5	22.3	508	90	5.7							
407A	211.87	196.55	19.65	92.77	19.65	98.02	93.63	161,603,119	82.26	512,898	28	Male	Phlegm and blood stasis obstruction	1 Year	Hushentongfengtai	Before treatment															
407B	144.58	131.07	13.11	90.66	13.10	97.88	92.87	105,880,882	81.39	458,053	28	Male	Phlegm and blood stasis obstruction	1 Year	Hushentongfengtai	After treatment															
408A	184.52	167.32	16.73	90.68	16.72	97.90	93.29	130,580,272	81.20	546,403	27	Male	Phlegm and blood stasis obstruction	2 Years	Hushentongfengtai	Before treatment															
408B	210.03	201.98	20.19	96.17	20.19	97.66	92.31	157,084,783	81.85	603,436	27	Male	Phlegm and blood stasis obstruction	2 Years	Hushentongfengtai	After treatment															
409A	179.36	163.58	16.35	91.20	16.35	97.92	93.47	132,984,967	81.43	524,958	48	Male	Phlegm and blood stasis obstruction	5 Years	Hushentongfengtai	Before treatment	6.66	144	234	25.7	20.5	410	95	3.8	4.98	2.07			6.4	6	1.6
409B	157.03	138.30	13.83	88.07	13.83	97.59	92.48	108,838,790	78.78	677,625	48	Male	Phlegm and blood stasis obstruction	5 Years	Hushentongfengtai	After treatment	7.25	142	239			267	91	4.3							
410A	173.61	157.98	15.80	91.00	15.80	97.95	93.37	126,329,465	80.01	446,197	45	Male	Phlegm and blood stasis obstruction	10 Years	Hushentongfengtai	Before treatment				13.5	14.6	380	110	3.3	5.28	4			5.25		
410B	172.80	157.11	15.71	90.92	15.71	97.96	93.53	124,513,752	79.27	557,369	45	Male	Phlegm and blood stasis obstruction	10 Years	Hushentongfengtai	After treatment	6.12	148	238	13.3	15.1	670	108	3.8	5.72	5.75	0.94	2.57	5.39	15	1.4
411A	196.84	182.72	18.27	92.83	18.27	98.08	93.58	142,432,108	79.77	1,064,749	35	Male	dampness-heat with blood stasis	3 Years	Tongfengtai	Before treatment															
411B	215.25	201.30	20.12	93.52	20.12	98.16	93.80	159,468,195	80.01	1,244,580	31	Male	dampness-heat with blood stasis	3 Years	Tongfengtai	After treatment															
C1	358.75	351.06	35.11	0.98	30.91	97.25	91.32	188,544,434	60.99	1,165,165	31	Male																			
C10	576.36	562.24	56.22	0.98	56.20	97.48	92.03	303,093,668	53.93	1,123,743	23	Male																			
C11	420.71	409.16	40.92	0.97	40.58	96.82	89.94	311,497,355	76.76	736,425	25	Male																			
C12	425.43	414.13	41.41	0.97	41.36	96.67	89.45	252,606,920	61.07	1,068,828	42	Male																			
C13	454.25	441.04	44.10	0.97	44.06	96.82	89.97	237,833,701	53.98	1,058,094	60	Male																			
C15	425.17	414.58	41.46	0.98	41.14	96.90	90.18	317,784,841	77.24	728,541	30	Male																			
C17	296.75	289.15	28.92	0.97	28.85	96.67	89.51	224,162,289	77.71	1,163,166	33	Male																			
C18	432.75	417.41	41.74	0.96	35.73	96.91	90.25	234,274,625	65.56	640,644	38	Male																			
C19	522.34	502.10	50.21	0.96	50.04	97.34	91.39	362,731,172	72.49	992,914	45	Male																			
C20	459.06	447.04	44.70	0.97	44.32	96.60	89.30	346,754,573	78.24	871,236	42	Male																			
C21	414.80	404.61	40.46	0.98	40.45	97.00	91.33	321,134,217	79.40	849,566	31	Male																			
C22	536.26	518.21	51.82	0.97	51.66	97.33	92.26	353,220,798	68.38	697,960	42	Male																			
C23	264.46	257.06	25.71	0.97	23.96	96.84	91.05	171,253,725	71.48	666,815	42	Male																			
C24	407.94	397.75	39.77	0.98	39.73	96.90	91.01	317,012,435	79.79	818,768	47	Male																			
C26	362.65	353.96	35.40	0.98	35.36	96.81	90.83	197,734,822	55.92	1,041,398	28	Male																			
C27	294.05	286.14	28.61	0.97	28.53	96.60	90.14	159,705,247	55.97	956,329	45	Male																			
C3	338.03	330.46	33.05	0.98	33.00	97.23	91.46	251,336,097	76.17	951,947	27	Male																			
C5	653.41	633.30	63.33	0.97	63.29	98.04	93.85	504,309,079	79.68	645,063	42	Male																			
C7	407.27	395.98	39.60	0.97	37.83	97.27	91.38	278,983,388	73.75	774,983	55	Male																			
C8	493.37	477.36	47.74	0.97	47.66	97.49	92.35	350,791,017	73.61	1,096,325	60	Male																			
C9	284.32	278.15	27.81	0.98	27.76	97.15	91.14	214,515,060	77.27	736,479	41	Male																			
Con1	202.67	184.99	18.49	91.28	18.49	97.95	93.27	144,734,998	78.26	1,192,589	35	Male																			
Con2	145.39	136.06	13.60	93.58	13.60	98.17	94.08	108,559,747	79.84	709,412	35	Male																			
Con3	224.62	208.96	20.89	93.03	20.89	98.18	93.81	169,118,764	80.98	927,961	40	Male																			
Con4	239.06	224.18	22.41	93.78	22.41	98.22	94.15	183,141,514	81.71	1,102,938	28	Male																			
Con5	203.02	188.77	18.87	92.98	18.87	98.12	93.72	152,140,522	80.62	1,219,270	38	Male																			
Con6	224.90	208.23	20.82	92.59	20.82	98.09	93.68	163,173,095	78.41	1,024,577	45	Male																			
Con8	229.92	214.52	21.45	93.30	21.45	98.17	94.04	165,137,806	77.00	1,376,387	36	Male																			
Con9	192.14	172.10	17.20	89.57	17.20	97.92	93.11	133,098,072	77.84	510,410	42	Male																			
