# Supplementary material for: Metagenomic Analysis of Gut Microbiome in Gout Patients with Different Chinese Traditional Medicine Treatments
Source: Evid Based Complement Alternat Med. 2022 Oct 10;2022:6466149. doi: 10.1155/2022/6466149 (PMC9576389; doi:10.1155/2022/6466149)
Supplement: Supplementary Materials — The present study contains 7 supplemental material files, including 3 supplementary figures and 4 supplementary tables; the legends are as followed: (1) Figure S1. Genus abundance and PCoA analysis for four treatment groups (please refer to Figure S1.docx). (2) Figure S2. Differential expressed gene and KOs pathway enrichment (please refer to Figure S2.docx). (3) Figure S3. Trend chart of other species abundance on phylum level in three groups (please refer to Figure S3.docx). (4) Table S1. Sequencing data summary (please refer to Table S1.pdf). (5) Table S2. Phylum annotation and relative abundance in three groups (please refer to Supplementary data.docx). (6) Table S3. Statistics of selected upregulation and downregulation samples (please refer to Supplementary data.docx). (7) Table S4. Biochemical indicators (please refer to Table S4.pdf). [file 6466149.f1.zip › 6466149.f1/TableS4 (2).pdf]

Sample_N	Hemameb	Hemoglob	Blood_pla	ALT	AST	UA	Cr	urea nitrog	FBG	Triglyceric	HDL	LDL	Cholestero	ESR	CRP
101A	_	_	_	40.9	29.9	558	67	3.6	5.09	5.24	0.71	2.74	4.81	_	_
101B	8.19	155	394	30.6	24.7	394	79	4.6	4.58	2.49	0.82	3.96	5.6	2	1
102A	12.3	130	385	18.6	12.3	_	90	5.1	6.34	_	_	_	_	41	53.6
102B	5.72	162	260	52.3	31.6	346	87	4.8	5.07	2.81	0.92	4.25	5.79	2	2.8
103A	_	_	_	_	_	487	86	2.4	5.01	1.37	1.4	3.81	5.36	_	_
103B	_	_	_	_	_	599	98	3.3		1.08	1.5	3.86	5.38	_	_
104A	19.6	100	467	_	_	_	_	_	_	_	_	_	_	_	_
104B	6.98	121	380	17.9	17.1	695	95	55	4.96	2.77	0.92	2.96	4.85	35	15.3
107A	_	_	_	16.9	20	378	113	4.6	_	_	_	_	_	_	_
107B	7.73	161	229	26	21.6	435	110	4.3	5.42	2.04	_	_	5.44	6	2.6
108A	8.98	164	315	34.7	24.5	600	81	4	4.95	1.81	_	_	5.2	2	1.5
108B	7.26	151	337	15.9	16.8	405	85	3.7	_	1.32	0.85	2.93	4.15	11	16.3
109A	7	139	272	12.5	22.5	180	93	6.7	_	_	_	_	_	51	5.1
109B	_	_	_	27	30.7	390	97	5	_	_	_	_	_	16	2.3
112A	5.95	159	286	89.4	64.1	616	103	5.3	5.21	1.61	0.94	2.86	4.34	15	11
112B	5.5	159	249	36	57.8	404	102	4.1		1.82	0.88	3.8	5.16	8	2.1
113A	5.87	163	254	23.2	21.4	461	120	5	6.07	_	_	_	_	_	_
113B	4.89	164	213	19.1	17.3	415	113	4	6.53	_	_	_	_	_	_
114A	4.69	133	74	22.9	19.8	238	129	4.9	4.89	0.81	1.48	1.78	3.46	18	61.2
114B	4.87	142	89	28.1	27.1	752	114	3.4	_	_	_	_	_	_	_
201A	6.1	148	298	28.9	26.7	248	86	4.5		1	1.19	3.6	5.23	_	_
201B	7.46	153	315	19.2	21.9	568	83	3.9	5.02	1.15	1.14	4.14	5.7	5	0.3
301A	8.05	149	294	14.3	13.5	572	87	5.8	5.04	2.27	1.03	5.23	6.6	3	1.1
301B	9.9	6.29	300	13.6	11.7	283	83	6.1	_	_	_	_	_	9	17.8
302A	8.71	122	419	11.2	15.7	262	53	4.3	6.67	3.66	1.23	3.35	5.66	70	10.8
302B	8.38	120	395	12.3	13.5	587	69	5.4	7.63	1.89	1.29	2.58	4.29	_	_
303A	4.07	146	155	18.2	27.3	426	85	2.8	5.19	2.87	0.82	2.3	3.96	14	1
303B	4.18	151	141	15.4	17.7	513	89	4		3.05	0.971	2.44	4.23	_	_
306A	7.48	162	259	64.9	36.9	303	79	2.4	5.52	4.31	0.88	3.29	5.38	1	0.9
306B	7.95	163	288	73.9	39.6	319	85	2.8	6.16	2.93	0.96	4.06	5.59	1	1.1
307A	7.09	171	241	26.4	21.5	655	99	3.5	5.52	1.92	0.96	4.32	5.73	11	7.4
307B	6.32	168	197	26.8	21.7	498	97	3.3	6.55	2.19	1.1	3.9	5.72	_	_
308A	7.85	137	351	17.4	18.9	491	76	4.6	_	4.61	1.2	4.97	6.72	10	5.3
308B	10.24	132	396	12.6	17.8	351	80	4.7	_	1.88	1.31	3.09	5.03	27	16.2
311A	4.96	5.11	224	10.8	16.6	328	94	3.8	6.15	4.7	0.81	3.43	5.92	7	1.9
311B	6.25	136	158	5.6	10.8	285	86	3.2	_	_	_	_	_	_	_
402A	_	_	_	20.7	17.8	393	93	3.6	_	_	_	_	_	_	_
402B	_	_	_	_	_	522	86	2.5	_	_	_	_	_	_	_
403A	_	_	_	28	19.7	431	84	5.1	_	_	_	_	_	_	_
403B	11.34	156	250	44	24	577	85	4.4	_	3.63	0.7	2.76	4.05	_	_
404A	6.55	159	199	29.7	26	536	76	2.2	_	2.17			4.19	2	0.8
404B				36.4	29.7	269	75	3.1	_	_	_	_	_	_	_
406A	6.05	170	242	31.7	23.1	740	108	4.4	4.64	2.82	0.99	3.73	5.52	4	0.8
406B	6.15	157	232	32.5	22.3	508	90	5.7	_	_	_	_	_	_	_
409A	6.66	144	234	25.7	20.5	410	95	3.8	4.98	2.07			6.4	6	1.6
409B	7.25	142	239	_	_	267	91	4.3	_	_	_	_	_	_	_
410A	_	_	_	13.5	14.6	380	110	3.3	5.28	4	_	_	5.25	_	_
410B	6.12	148	238	13.3	15.1	670	108	3.8	5.72	5.75	0.94	2.57	5.39	15	1.4
